# Building a Relationship between Robot Characteristics and Teleoperation User Interfaces

**DOI:** 10.3390/s17030587

**Published:** 2017-03-14

**Authors:** Michael Mortimer, Ben Horan, Mehdi Seyedmahmoudian

**Affiliations:** School of Engineering, Deakin University, Pigdons Rd, Waurn Ponds, Victoria 3216, Australia; ben.horan@deakin.edu.au (B.H.); mehdis@deakin.edu.au (M.S.)

**Keywords:** teleoperation, User Interface, Robot Operating System (ROS), Matlab, Unified Robotic Description Format (URDF), Sematic Robotic Description Format (SRDF)

## Abstract

The Robot Operating System (ROS) provides roboticists with a standardized and distributed framework for real-time communication between robotic systems using a microkernel environment. This paper looks at how ROS metadata, Unified Robot Description Format (URDF), Semantic Robot Description Format (SRDF), and its message description language, can be used to identify key robot characteristics to inform User Interface (UI) design for the teleoperation of heterogeneous robot teams. Logical relationships between UI components and robot characteristics are defined by a set of relationship rules created using relevant and available information including developer expertise and ROS metadata. This provides a significant opportunity to move towards a rule-driven approach for generating the designs of teleoperation UIs; in particular the reduction of the number of different UI configurations required to teleoperate each individual robot within a heterogeneous robot team. This approach is based on using an underlying rule set identifying robots that can be teleoperated using the same UI configuration due to having the same or similar robot characteristics. Aside from reducing the number of different UI configurations an operator needs to be familiar with, this approach also supports consistency in UI configurations when a teleoperator is periodically switching between different robots. To achieve this aim, a Matlab toolbox is developed providing users with the ability to define rules specifying the relationship between robot characteristics and UI components. Once rules are defined, selections that best describe the characteristics of the robot type within a particular heterogeneous robot team can be made. A main advantage of this approach is that rather than specifying discrete robots comprising the team, the user can specify characteristics of the team more generally allowing the system to deal with slight variations that may occur in the future. In fact, by using the defined relationship rules and characteristic selections, the toolbox can automatically identify a reduced set of UI configurations required to control possible robot team configurations, as opposed to the traditional ad-hoc approach to teleoperation UI design. In the results section, three test cases are presented to demonstrate how the selection of different robot characteristics builds a number of robot characteristic combinations, and how the relationship rules are used to determine a reduced set of required UI configurations needed to control each individual robot in the robot team.

## 1. Introduction

Robots provide a range of benefits for today’s society, including important applications such as Urban Search and Rescue (USAR) [1], medical [2], environmental [3], transportation [4], and smart agriculture [5]. The Robot Operating System (ROS) was developed to provide roboticists with a large scale, open-source research platform to help simplify the integration of robotic systems [6]. Arguably the biggest advantage over other robotic frameworks is the implementation of a peer-to-peer network topology and microkernel environment. This peer-to-peer approach, based on the TCP/IP protocol, allows systems to be interconnected within heterogeneous networks. This, combined with the built-in message description language used to describe common data types, creates a simple protocol for robotic systems to communicate through topics using a simple publish and subscribe method. ROS supports a large number of different robots [7,8,9,10,11] and includes a variety of important robot independent packages [12,13,14,15,16]. This provides developers with a wide range of support and functionality, able to be easily deployed to a ROS supported robot.

While autonomous robots commonly provide a robust solution for structured environments and well-defined tasks, human-in-the-loop control is typically deployed for non-deterministic and unstructured environments. In robotics, teleoperation refers to the control of a robot over a telecommunication medium, typically at distance, and the effectiveness of the User Interface (UI) is integral to teleoperation success. UI design is crucial in any application requiring Human Computer Interaction (HCI), and is particularly important in teleoperation, where the teleoperator is required to perceive the robot’s operating environment and control the robot appropriately. There are a range of technical challenges which need to be overcome to develop teleoperation functionality and [17] lists many of these, including the limited Field of View (FoV) of camera feeds, lack of depth perception, difficulty inferring the orientation of a remote robot, ability to control multiple cameras and sensors, poor frame rates, time delays, and challenges in motion control.

The increasing capability of robot semi-autonomy can lessen the extent to which such issues impact on purely teleoperated robots, i.e., those controlled only by the teleoperator with no supporting autonomy. Employing robots with some degree of autonomy, known as semi-autonomous robots, in teleoperation tasks can be beneficial. An example of this is the ability to accept high-level waypoints from the teleoperator, but where the robot can autonomously navigate an environment while avoiding obstacles. In such a scenario it may also be desirable to allow the teleoperator to intervene and override the autonomous behavior of the robot; however, considerations such as time delay may not make this possible. Where certain semi-autonomous robot functions can be relied on, semi-autonomous teleoperation can lessen the demand on the teleoperator for low-level tasks and free them up for higher-level decision making tasks such as managing a team of robots [18]. Supervising a team of semi-autonomous robots, which can be considered supervisory control, presents the teleoperator with different challenges to those of lower-level tasks like the motion control of a single mobile robot. One such challenge when performing supervisory control of a robotic team is maintaining situational awareness when switching between different teleoperation controls [19].

ROS has the ability to cater for a large number of supported robots using custom packages [20]; however, it doesn’t provide a single or standard approach to teleoperating different robots or heterogeneous robot teams. Instead, distinct packages such as those presented in [12,13,21] provide dedicated interfaces for achieving a particular type of teleoperation control for certain robots. RViz [22] is a popular ROS package that provides an interface for viewing different sensory information such as camera vision, laser scan, and other point cloud data from different robots using the common message types available in ROS. While such packages have been widely used, they don’t provide a single teleoperation system able to support the teleoperation of different robots, or even robot teams as would be required for applications such as USAR [19]. In order to achieve such a teleoperation interface, and in particular one dynamic and responsive to switching between controlling different robots within a team, a different approach to teleoperation UI design is required.

A list of works evaluating the teleoperation of robot teams is outlined in [19]. Based on the findings and those of previous works, it is suggested that a single teleoperator typically shouldn’t control more than 8–12 semi-autonomous robots. Unfortunately, much of the work dealt with homogenous robot teams or heterogeneous robot teams comprised mainly of the same robot type, e.g., only Unmanned Ground Vehicles (UGV). In [23], real-world teleoperated robot team experiments were undertaken and the work discusses how human-robot interaction is the current barrier to more successful human-robot teaming. While trust in autonomy, as one consideration in human-robot interaction, is listed as a major challenge to successful teleoperation of robot teams, it should be noted that specific UIs were required for different robot types, such as in the third trial for UAV and UGV robots. This type of situation requires teleoperators to adapt to different UIs and presents the opportunity to develop a different approach. Such an approach could provide less variability in the UI and provide transferable skills through the reuse of UI components which perform similar teleoperation functions for the same or similar robot types, such as two different UAVs. Robot specific UIs for each different robot model of the same type is likely to affect a teleoperator’s situational awareness and increase the risk of operator overload by exposing the teleoperator to a large amount of information. Operator overload could be reduced by the reduction of the number of different UI configurations the teleoperator needs to be familiar with in order to contribute to improving interoperability across robots that have the same or similar functionality, such as might be encountered if an operator was required to teleoperate different UGVs. In [24] robots are connected through the internet so as to share information and complete tasks through autonomous collaboration. The work details a “*skill abstraction layer*” which attempts to identify the common robot functions that are hardware-independent, providing a standard approach to the autonomous operation of heterogeneous robots.

To summarize, a gap in the literature is identified as: (a) the ability for the teleoperation system to identify robots within a particular team with similar capabilities in order to potentially allow the same UI configuration to be used for the similar robots, thereby reducing the number of different UI configurations the teleoperator needs to be familiar with; (b) provision of a single or systematic approach to teleoperation UI design for individual robots within heterogeneous robot teams; (c) improve approaches for swapping teleoperational control between different robots within a heterogeneous robot team; and (d) a systematic approach to generating teleoperation interfaces including motion control, sensor presentation methods, and UI components, for controlling individual robots consisting of similar or the same functionality. To take a step towards addressing these challenges, as well as more generally towards a standard approach to systematically identifying a minimal set of UI configurations able to control the different robots in a heterogeneous robot team, this paper introduces the concept of relating robot characteristics to required components of the teleoperation UI. Throughout this paper, a UI configuration refers to the components required to be part of the UI in order for the teleoperator to control the necessary functions (such a motion control) and to receive the necessary information from the required sensory of the individual robot. An example could be where a UI configuration for teleoperating a UAV may include flight controls and display of 2D camera feed. Given that UAVs often have similar functionality it is worthwhile to consider that another UAV in the team may be identical or share many of the same characteristics as the above mentioned UAV. In this case it is worthwhile considering if the same UI configuration could be used for teleoperation of each UAV respectively. This is considered with the view that by not having different UI configurations for the two same or similar UAVs, the number of different UI configurations the operator needs to be familiar with can be reduced. Because the commonality across robots is most apparent across robots of the same type (i.e., UAV), this paper considers reducing the different UIs across the same robot type, but lays the foundations for later work to consider common characteristics across different robot types such as humanoid and UAV.

While the layout and physical setup of the UI extend beyond the scope of this work, the process for generating UI configurations provide the necessary information regarding what needs to be present in the UI. The approach presented in this paper identifies the common robot characteristics across different robots of the same kinematic type using ROS metadata, and in doing so attempts to reduce the number of different UIs configurations required to control similar robots. Because the approach uses ROS metadata, it can be later applied during teleoperation to simplify the determination of UI configurations for the teleoperator’s current situation.

The approach presented in this paper is implemented as a Matlab toolbox, and involves a three step process. The first step of the process requires users to specify a set of rules relating common robot characteristics (the robot characteristics informed by ROS metadata) and teleoperation UI components. This first step is only required for initial setup or where rules need updating; once relationship rules are created, they are reused for different robot teams. Given a defined relationship rule set, the second step is where selections of robot characteristic for each robot type, e.g., UAV, are made. The third and final step is where, using the relationship rules and robot characteristic selections for the given robot team, the toolbox automatically determines the number of different teleoperation UI configurations required to be designed for each robot type to teleoperate individual robots within the particular robot team.

The remainder of the paper is structured as follows: The background to ROS and the relationship to teleoperation UIs are presented in Section 2. The design and development of the proposed approach, including the Matlab toolbox, are presented in Section 3. In Section 4, three test cases are selected in order to evaluate the performance of the developed Matlab toolbox in determining the number of UI configurations. Finally, the Conclusion and Future Works will be presented in Section 5.

## 2. Background

### 2.1. The Robot Operating System

A powerful feature of ROS is the ability to describe individual robots using description languages and represent sensory information with its standard message types. In particular, this includes the Unified Robot Description Format (URDF), Sematic Robotic Description Format (SRDF), and sensor message definitions. The URDF, SRDF and sensor messages contain a large amount of information about robot characteristics able to be exploited for different applications including teleoperation. In ROS, robots are commonly described using the URDF standard [25].

The URDF is an Extensible Markup Language (XML) specification, and in ROS is used to provide information about the dynamics, kinematics, sensor descriptions, visual representation data, and collision models for a given robot. To describe a robot in ROS, the URDF descriptor contains seven XML elements: link, joint, transmission, sensor, model, model state, and Gazebo properties. The first six elements contain descriptive information able to be used by any package within ROS. The last element, Gazebo, relates to properties specific to simulation, such as damping and friction, for use in the Gazebo simulator [26].

The URDF has some limitations, such as the inability to describe a serial chain of joints, commonly used to represent a robotic arm or manipulator. The SRDF is similar to the URDF but overcomes such limitations, and contains nine XML elements; robot, group, group state, link, joint, chain, end effector, virtual joint, disable collisions, passive joint, sphere, and link sphere approximation. A well-known example using the SRDF is the *MoveIt!* mobile manipulation package introduced in [12]. In *MoveIt!*, the setup wizard allows the creation of an SRDF formatted file for a given robot where one doesn’t already exist. *MoveIt!* then uses a robot’s SRDF description to provide interactive teleoperation manipulator control. Just like in *MoveIt!*, the SRDF information can be utilized in a range of teleoperation packages. Aside from the URDF and SRDF description formats, ROS also utilizes a message description language based on common data types for communicating relevant information. In the ROS message description language, each line represents a data field and corresponding name as shown below (1).

(1) $$\begin{array}{lll} \mathrm{datatype}1\;(e.g.,\mathrm{int}8,\mathrm{uint}32,\mathrm{string}) & & ...\mathrm{name}1\;(e.g.,x)\\ \mathrm{datatype}2\;(e.g.,\mathrm{float},\mathrm{time},\mathrm{bool}) & & ...\mathrm{name}1\;(e.g.,y) \end{array}$$

In ROS, single ROS messages are stored in separate files denoted by *“.msg”* file extension. The ROS *“sensor_msgs”* package contains a range of ROS messages each describing data for a particular sensor, such as a camera, laser scanner, joystick, or Inertial Measurement Unit (IMU). An application using these ROS sensor messages is discussed in [21] where this information is used to visualize different sensor information.

### 2.2. ROS Metadata and Teleoperation User Interface

When designing an interface for the teleoperation of robots, it is important to consider the relationship between robot capabilities; in particular those relevant to the required task, and the configuration of the teleoperation UI. It is also important that the teleoperator is provided with enough sensory information, in an intuitive manner where possible, such that adequate telepresence is achieved without overloading the teleoperator with too much information. Sensory information about the remote environment obtained by a robot is commonly presented to the teleoperator visually; however, interaction with other sensory modalities such as haptic and auditory is less common but still possible if available hardware permits.

**Figure 1:**
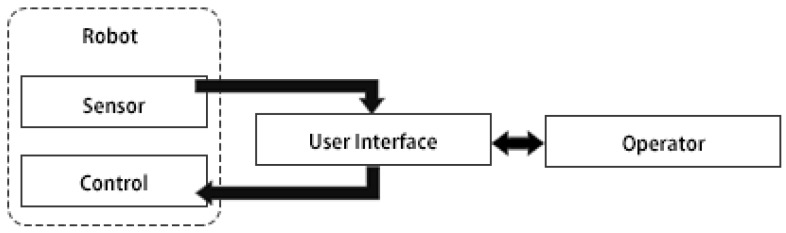
High-level information flow for teleoperation applications.

Figure 1 depicts a high-level overview illustrating the typical flow of information between the human and robot during teleoperation. As shown, sensory information obtained by the robot is presented to the teleoperator by way of the UI, and control commands are sent to the robot by the teleoperator through their interaction with the UI. As is apparent, the UI is a critical component of the teleoperation process. The UI needs to provide the teleoperator with necessary information regarding the robot and its environment, as well as the ability to provide commands to the robot. This work proposes an approach allowing users to specify rules representing relationships between certain robot characteristics (both relating to sensors and control commands) and components of the UI used by the teleoperator to command the robot team. The developed toolbox then determines the number of different UI configurations required to teleoperate each individual robot within a team. It is suggested that such work is a step towards providing a systematic approach to teleoperation UI design by identifying a reduced set of UI configurations. Additionally, because different robots of the same robot type can share the same or similar robot characteristics, the number of different UI designs required to teleoperate a robot team can be reduced. For example, consider a robot team comprising of three different UAVs, each from a different manufacturer, and each having a single 2D camera and a specific set of direct flight controls for basic teleoperation. Currently it is likely that the three UAVs each have different teleoperation UIs, despite having similar capabilities, i.e., all providing the teleoperator with a view of the remote environment by a robot-mounted 2D camera, and being controlled using direct flight control. Currently, in order for a teleoperator to control this team of three UAVs they would need to be familiar with the three different teleoperation UIs. Given the similarities between a team of different robots of the same type, i.e., the team of three UAVs, it is possible to provide a single teleoperation UI configuration that could be used for all three UAVs which supports the teleoperator to transfer their skills between different robots, improving interoperability.

Virtual Reality (VR) and other immersive technologies, while not a focus of this work, may provide benefits when teleoperating robot teams using traditional peripherals such as monitors, keyboards, and joysticks. Consider a UAVs having three 2D cameras onboard, and each camera would commonly be viewed using an individual monitor, or a single monitor by either swapping camera displays using devices such as keyboard and mice or by tiling video feeds. A VR system including a tracked Head Mount Display (HMD), and hand controllers such as the HTC Vive or Oculus Rift, could provide a 360° reconfigurable virtual environment consisting of virtual displays of any size and orientation for each of the cameras and able to be easily moved or adjusted. Doing so could overcome limitations associated with physical workspaces such as a control room which may not be able to be quickly reconfigured as required.

### 2.3. Relationship between ROS Metadata and Teleoperation User Interface

In robotics, a robot type is typically described using terminology well-known in the robotics field and assigned using knowledge and human intuition. Through knowing a robot’s type, a teleoperator can make educated assumptions about the robot’s capabilities, sensory information, and motion control options. This allows teleoperators to mentally prepare for teleoperating the selected robot and for the type of task it would normally perform. Examples of such tasks for a particular robot type include UAVs, which often provide teleoperators with bird’s eye view of a remote environment and mobile manipulators which have the ability to traverse ground terrain and interact with objects in the remote environment [27].

**Figure 2:**
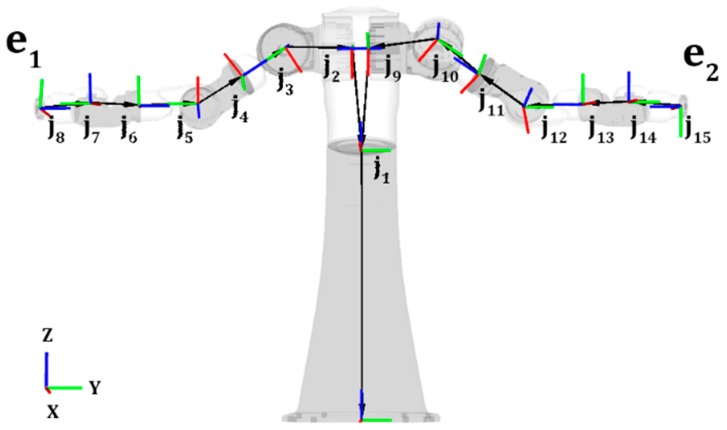
Torso robot joint and link tree using Motoman SDA20D URDF in RViz [22]. $J_{i}$ represents joints, $e_{i}$ represents end effectors.

A teleoperation UI able to automatically identify a robot’s type can provide teleoperators with valuable information aiding them in determining the robot’s capabilities. This determination of robot type requires type definitions allowing robots to be grouped based on known terminology, as explained above. As discussed in the previous section, ROS provides kinematic information within the URDF describing individual robots, in particular the robot’s joint and link tree. Patterns within URDF joint and link trees, such as that shown in Figure 2, can be used to determine a robot’s type based on their kinematic configuration. For example, a humanoid robot consists of two arms, two legs, and a head. A torso robot has similar characteristics to that of a humanoid, with the main difference being not having legs. In the approach presented in this paper, Robot Kinematic Type is one of the four robot characteristics able to be selected using the toolbox and is used to group robots with similar kinematic types.

To complete tasks effectively, the teleoperator requires information about the remote environment obtained through sensors on the robot. A single robot can consist of a vast array of onboard sensors, and these are typically presented within a UI using visual [28,29], audio [30], and sometimes force feedback [31] presentation methods. If the type of sensor is known in advance, then the best suited presentation method for that sensor can be determined and remote information effectively communicated to the teleoperator in a consistent manner.

**Figure 3:**
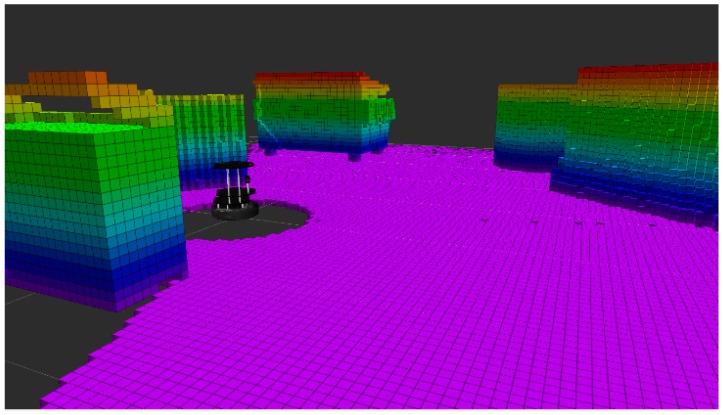
Visualisation of simulated TurtleBot Kinect sensor point cloud in RViz [22], post-processed using OctoMap [15] method available in ROS OctoMap package.

In ROS, information about a robot’s onboard sensors is generally contained within two different forms of metadata: the URDF and sensor messages. The URDF uses the XML sensor element to define the location of sensors within the robot’s kinematic tree, and also provides basic descriptive information about each listed sensor. A ROS-supported robot typically provides a list of topics that publish messages containing information about the robot including sensors. Looking at a robot’s topic list shows its messages currently being published in ROS, and by searching these topics for a particular robot, the number and sensor types for an individual robot can be ascertained. Once the sensor information is known, then the method to present the data to the teleoperator can be assigned. Figure 3 shows an example of how point cloud data can be visualized. We obtained this data from a simulated Kinect device on a virtual TurtleBot and by using a published “*PointCloud2*” message definition described in the ROS “*sensor_msgs*” package. The point cloud data was then post-processed using the OctoMap method from [15] available in the OctoMap ROS package to create a stable voxelized representation of the simulated remote environment. This was conducted as part of this paper’s exploration into sensors able to share the same visual presentation methods.

Teleoperation requires the teleoperator to communicate the required commands to the remote robot. Depending on the requirements of a given task and the robot’s capabilities, the teleoperator can either control the robot through pure teleoperation or with the robot exercising some degree of autonomy. Regardless of the level of autonomy, with the exception of a fully autonomous robot, the teleoperation UI is used to communicate with the remote robot so that required tasks can be completed.

ROS-supported robots are able to be teleoperated using their respective teleoperation packages that can commonly be identified by a “*teleop*” postfix. These packages are typically created by the developers and researchers responsible for the robot’s implementation into ROS. Teleoperation packages typically comprise information about the different teleoperation strategies such as driving, flying, or waypoint control used for teleoperating the specific robot. Unfortunately, there is currently no standard approach to representing teleoperation information for different robots within ROS. As such, in order to gain an understanding of typical teleoperation approaches for different types of robots, a survey on currently supported ROS robots and other common teleoperation techniques was conducted and listed in Table 1.

## 3. Materials and Methods

### Matlab Toolbox

The previous section discusses how ROS metadata contains different robot information including kinematics, the number and types of sensors, and motion control strategies. This section explores how this metadata is used to develop a Matlab toolbox for automatically determining and reducing the number of different teleoperation UI configurations required to control individual robots within a robot team. This process considers each separate robot type one at a time, and identifies the number of different teleoperation UI configurations required. As discussed earlier, the similarity in function of individual robot types is considered to look for opportunity to provide the same UI configuration for similar robots, and this is based on the relationship rules and selected robot characteristics as defined by the user. ROS metadata is used in two different ways, the first being to inform the choice of different robot characteristics able to be selected when using the toolbox, and the second is to identify a teleoperated robot’s currently selected characteristics so that the required UI configuration can be provided during teleoperation. The former occurs during use of the Matlab toolbox, and the latter in real-time. Real-time use extends beyond the scope of this particular paper, but would be where ROS-supported robots are connected to a teleoperation system, and different UI configurations are automatically assigned using the robot’s ROS metadata. The layout and physical orientation of the UI is outside of the scope of this paper, but the UI configuration provides essential information about the components needed within the UI.

The toolbox determines the number of different UI configurations required to operate the robot team, considered type-by-type, as determined using rules defining the relationship between robot characteristics (informed by ROS metadata) and UI components. The number of UI configurations determined by the toolbox provide at least one UI configuration to be used by each individual robot within the team. By assigning the same UI configurations to the same or similar robots, the number of overall UI configurations can be reduced for each robot type where appropriate, and the overall robot team.

An example of where the same UI configuration cannot be used for two robots of the same type would be where a robot team comprises both a UGV with direct driving control and 2D camera vision, and another a UGV that has waypoint control and 2D camera vision. Because they require different motion control inputs, i.e., have different Robot Motion Control Characteristics, using the same UI configuration is not possible.

Information regarding the types of sensors onboard a robot, and consequently the components required in a UI configuration can be obtained from ROS metadata. For instance, 2D and 3D cameras could both use a visual display as the necessary UI component to present the camera vision, whereas a 360° camera would likely be better served by an appropriate geometry such as a sphere to display the 360° vision correctly. Using the same visual presentation methods for similar sensors across different robots is another example of how the same or similar robot characteristics can be presented by the same UI configurations. These relationship rules are updatable, allowing for future changes to the type of sensors and motion control strategies that may be introduced by the ROS open-source and robotics’ communities.

**Figure 4:**
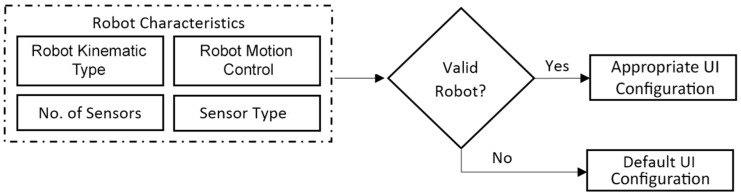
Flowchart overviewing the Matlab toolbox teleoperation UI configuration assignment based on robot characteristic.

Once relationship rules are defined, selections specifying the characteristics of robots and those that describe a particular robot team can be made using the Graphical User Interface (GUI) shown in Figure 8. These selections define robot characteristic combinations, referred to as valid robots, which dictate the required number of different teleoperation UI configurations that need to be determined to represent each different robot characteristic combination within the team. When different robots of the same Robot Kinematic Type share the same or similar robot characteristics, the system can reduce the number of teleoperation UI configuration designs required using previous determined relationship rules by assigning the same teleoperation UI configuration. There are four robot characteristics to be specified, as shown in Figure 4, and the choice of these four characteristics and their constituents were informed through surveying ROS supported robots and other available data.

When the selections specifying the robot characteristics have been made, a list containing all valid robot combinations matching these characteristics is defined. Figure 4 shows how all valid robots are assigned a UI configuration. This assignment is based on the rules describing the relationship between robot characteristics and components of the UI. For example, all robots that are of a manipulator Robot Kinematic Type a 2D camera Sensor Type and end effector Robot Motion Control with less than 5 sensors can be assigned with the same UI configuration. The default UI configuration is assigned when an invalid robot configuration is encountered. An invalid robot occurs when the system is presented with a robot that has an unknown characteristic combination, and therefore is unable to assign a known UI configuration. The default UI configuration contains all possible UI components that the system has the ability to provide, but is unable to assign an appropriate UI configuration as opposed to a valid robot. An example would be presenting the system with characteristic definitions described in Table 2 with a quadruped that had 360 camera vision. This system described in Table 2 would not be able to determine a quadruped Robot Kinematic Type, so therefore it can’t assign a known UI configuration; in this case a default UI configuration would be assigned. This UI configuration would search the invalid robot for known characteristics such as the 360 camera vision or other characteristics in Table 2, and provide relevant UI components. It is intended that in future work, when a teleoperator is switching between robots or even characteristics within the same robot during teleoperation, that the system can assign the required UI configuration if the robot is considered valid. These individual UI configurations for the connected robots will be based on the robot’s characteristics identified by its ROS metadata as presented in this paper.

**Figure 5:**
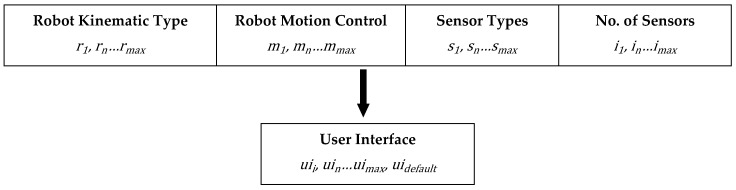
Matlab toolbox input and output formats.

Each UI configuration is associated with a whole number value in consecutive order with the default UI configuration assigned last. The data representing each robot characteristic needs to be formatted appropriately so that it can be represented numerically, with the numerical representation of each characteristic providing the required range. The input and output formats for each of the four robot characteristic types are shown in Figure 5 where $r_{n}$ represents Robot Kinematic Type, $r_{\max}$ the maximum number of Robot Kinematic Types, $m_{n}$ the method for Robot Motion Control, $m_{\max}$ the maximum number of methods for Robot Motion Control, $s_{n}\;$the Sensor Type, $s_{\max}$ the maximum number of Sensor Types, $i_{n}$ the No. of Sensors, $i_{\max}$ the maximum number of No. of Sensors, $ui_{i}$ appropriate UI configuration, ${\mathrm{ui}}_{\max}$ the maximum number of appropriate UI configurations, and ${\mathrm{ui}}_{\mathrm{default}}$ represents a default UI configuration assigned to invalid robot characteristic combinations.

**Figure 6:**
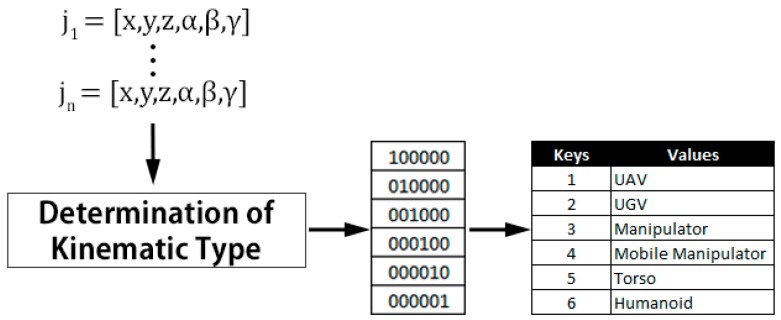
Formatting Robot Kinematic Type data, $j_{i}$ represents robot joints, x, y, z represent joint locations, and α, β, γ represent robot joint rotations.

In this work, the Robot Kinematic Type characteristic has six possible selections: UAV, UGV, Manipulator, Mobile manipulator, Torso, and Humanoid. These Robot Kinematic Types were chosen based on surveying common robots and considering common terminology used, listed in Table 1 (in results section below). It is important to note that this list is not fixed and could be adjusted to include different Robot Kinematic Types, such as quadruped robots. The specified Robot Kinematic Type is used by the relationship rule base to configure a smaller set of corresponding UI configurations for a particular Robot Kinematic Type due to their kinematic similarities. For example, all UI configurations assigned for robots that are of the UAV Robot Kinematic Type may include a virtual cockpit, while for a UGV’s the UI configuration might include a virtual driver’s seat. The Robot Kinematic Type choices are determined using kinematic information and robot type definitions that are then formatted into a numerical range using an associative array as depicted in Figure 6. While this list currently includes six different Robot Kinematic Types, it could be reconfigured to include extra Robot Kinematic Types through changing the robot definition types. For example, if a particular robot team included quadruped robots then its unique kinematic structure could be defined within the robot type definitions and listed as a possible Robot Kinematic Type.

The Robot Motion Control characteristic represents different motion control methods such as direct flight or waypoint controls, defined such that each method requires a component within the UI. Robot Motion Control may be applicable across different Robot Kinematic Types, such as individual joint control which could be used for controlling both Humanoid and Torso Robot Kinematic Types. Similar to the Robot Kinematic Type, the Robot Motion Control characteristic is also represented by an associative array after being converted to numerical values of an appropriate numerical range.

**Figure 7:**
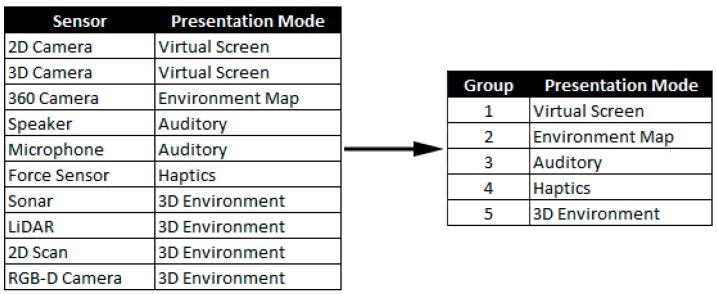
Grouping Sensor Types into sensor presentation groups using rules determined in this work.

As part of defining the rules representing the relationship between robot characteristics and components of the UI, Sensor Types requiring similar presentation methods can be grouped together (Figure 7). The presentation mode (right hand side of Figure 7) for sensor groupings represents the UI component which will be provided if a particular Sensor Type is specified in the robot characteristics. An example is 2D and 3D cameras that require similar sensor presentation methods, the mono and stereoscopic difference withstanding, and can both be displayed within a visual display in the UI configuration.

The relationship rules are used to implement logic based on the specified robot characteristics. The No. of Sensors characteristic specifies the maximum number of sensors onboard any given robot included in the selected Robot Kinematic Type. Considering the No. of Sensors characteristic, they can be clustered where a large amount is present on an individual robot. Clustering the sensors into separate clusters, as detailed in Equation (2), can reduce the physical space required to represent the UI components corresponding to the sensors. Doing this can be thought of as analogous to the use of folders in Microsoft Windows to group files of a common theme to save UI space. Using (2), if an individual robot is deemed to have no more than fifty sensors then there will always be five or less components referencing them in the teleoperation UI, represented either individually or as clusters. For example, if the No. of Sensors on a robot was three, they would be represented individually within the UI; whereas for a robot containing ten sensors, they would be clustered into two individual clusters. If a robot has more than the maximum No. of Sensors characteristic, then it is considered invalid and a default UI configuration is assigned as per Figure 4. While the design of the number of UI configurations determined using the Matlab toolbox is beyond the scope of this paper, future work could include different techniques for the organization of clusters such as alphabetically, most commonly used, or even by the amount of data needing to be transmitted. For example, a video UI component could be differentiated from an auditory component based on the difference in the amount of data required to be transmitted:

(2) $$g(i)=\;\{\;\begin{array}{cc} i & 1<i\leq 5\\ ⎡i/2⎤ & 6<i\leq 10\\ ⎡i/3⎤ & 11<i\leq 15\\ ⎡i/5⎤ & 16<i\leq 25\\ ⎡i/10⎤ & \;25<i_{\max} \end{array}$$

where g(i) represents the number of clusters rounded up to the nearest whole number, i represents the No. of Sensors robot characteristic, and $i_{\max}$ represents the maximum No. of Sensors.

**Figure 8:**
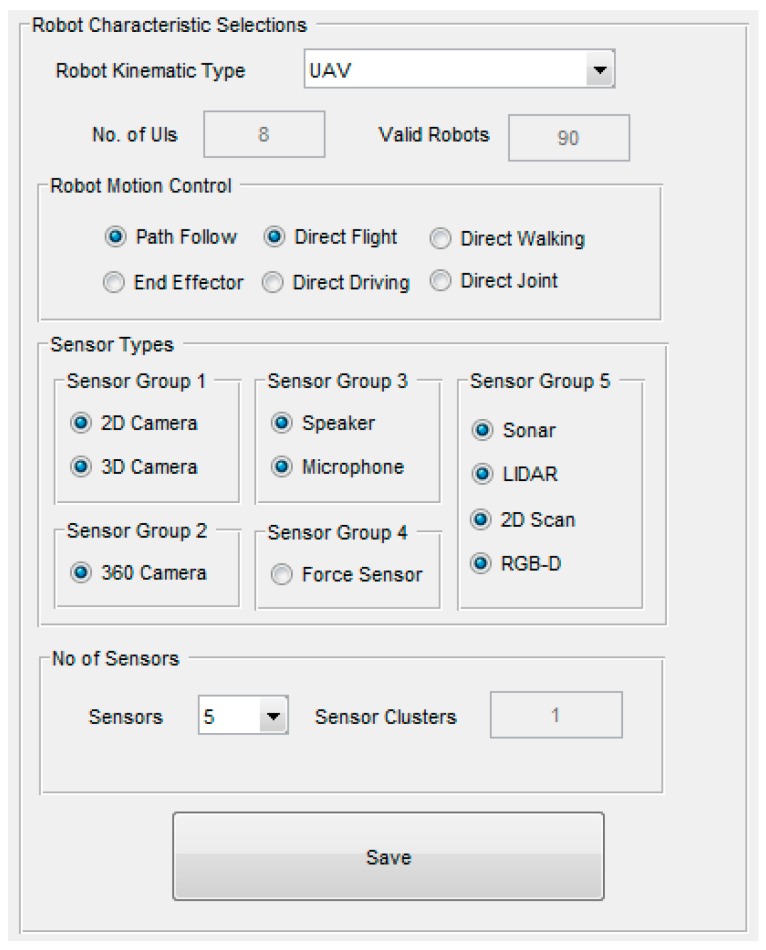
Matlab toolbox characteristic selections.

Once the four robot characteristics have been specified using the toolbox GUI (Figure 8), the toolbox determines all possible UI configurations based on the previously defined rules. For each possible robot characteristic combination for all individual robots with the described robot team, this UI configuration list can be searched to find the required UI configuration for a particular characteristic combination, for example flying a UAV with waypoint control and viewing a 2D camera view. The process of finding the corresponding UI configuration from the list and displaying it to the teleoperator can be performed in real-time during teleoperation. In fact this is the main benefit of using ROS metadata to identify different robot characteristics during teleoperation, as this allows the system to identify these robot characteristic changes when switching between different robots within a team. For example, consider a very small robot team that consists of two robots, a UAV and UGV. Now let’s consider the UAV has flight and waypoint Robot Motion Controls and a single 2D camera as Sensor Type, while the UGV consists of drive controls and a Red Green Blue-Depth (RGB-D) camera Sensor Type. Using the proposed system, these robot characteristics would be represented by three UI configurations; first configuration would be the UAV with flight control and 2D camera view, then the UAV with waypoint control and 2D camera, and finally UGV with drive control and RGB-D camera. Both robots in the team have less than five sensors, therefore sensor cluster is the same. Now during real-time operation when the teleoperator switches between the robots, or even the Robot Motion Controls on the UAV, the system will provide the corresponding UI configuration using the current characteristic combination as selected by the teleoperator. While the process of finding the corresponding UI configuration from the list and displaying it to the teleoperator can be in real-time, the focus of this paper is on determining the number of different UI configurations required to be designed for use by the teleoperator to teleoperate different robots within the team when using the system.

The number of different UI configurations are reduced using the same teleoperation UI configuration for robots within the same Robot Kinematic Type that have the same or similar robot characteristics. The degree to which the UI configurations can be used for different robot teams depends on the way in which the relationship rules are specified; but by reducing the number of different UI configurations required, it can contribute to reducing both teleoperation UI design and development time, as well as providing transferable skills for interoperability of different robots. This consistency aims to help improve switching teleoperational control between different robots within a team by sharing the UI configurations, such as displaying 2D cameras, and to improve the teleoperator’s overall situational awareness.

Figure 8 shows the toolbox GUI used to select robot characteristics and the selections shown are for the UAV Robot Kinematic Type. The UAV characteristic selections shown include waypoint and direct flight Robot Motion Controls. This selection was made based on the assumption that a UAV may support waypoint and/or direct flight Robot Motion Control methods. In terms of Sensor Types, all types except Sensor Group 4 (force feedback) have been selected, constituting four sensor groupings as discussed earlier. The maximum No. of Sensors of five was specified, and given (2) this means that they will be individually represented within the UI configurations.

As discussed earlier, once these selections are made for the selected Robot Kinematic Type, and the save button pressed, the toolbox determines the number of UI configurations required. The toolbox first determines all valid robot combinations based on robot characteristics selections, after which the number of different UI configurations required to control individual robots within the team is determined using relationship rules. In the case of Figure 8, the toolbox determined ninety valid UAV combinations and requires eight different teleoperation UI configurations. The toolbox keeps a system total of both valid robot combinations and different UI configurations as each Robot Kinematic Type are defined and save button pressed. At the end of robot characteristic selections for all required Robot Kinematic Types, the total number of UI configurations required are determined for the particular robot team.

## 4. Results and Discussion

The relationship between UI components and robot characteristics in the Matlab toolbox needs to be defined before the selection of characteristics defining the robot team, and is done by defining the relationship rules. In order to provide logical definitions and inclusions for the definition of the characteristics able to be selected (summarized in Table 1), previous research was surveyed and ROS metadata analyzed. This list of selections is not fixed and can be adjusted to include different Robot Kinematic Types, Sensor Types, and Robot Motion Control Methods. Using the definitions listed in Table 1, ranges are assigned to the Robot Kinematic Types, Robot Motion Controls, and Type of Sensors characteristics. The relationship rules assigned to the last robot characteristic, No. of Sensors, align with the five possible cluster arrangements in (2). In relation to this paper, the total number of possible teleoperation UI configurations can be determined using the following ranges: $r_{\max}\;=\;6$, $m_{\max}\;=\;6$, $s_{\max}\;=\;5$, and $i_{\max}\;=\;5$, as shown in (3). This value will always be less than all valid robot characteristic combinations due to the reduction obtained by similar robot characteristics of the same Robot Kinematic Type, resulting in the same UI configuration used for more than one robot characteristic combination:

(3) $${T\;=\;r}_{\max}{\;m}_{\max}{\;s}_{\max}{\;i}_{\max}\;=\;900$$

where T represents all possible UI configurations, $r_{\max}$ the maximum number of Robot Kinematic Types, $m_{\max}$ the maximum number of Robot Motion Controls, $s_{\max}$ the maximum number of Sensor Types groups, and $i_{\max}$ the maximum number of No. of Sensor.

In order to show the benefits of the approach embedded in the Matlab toolbox, three test cases are explored as described in Table 2. Each case successively increases the number of Robot Kinematic Types included and corresponding Robot Motion Control methods, Sensor Types, and the No. of Sensors characteristics selected. These characteristic selections were chosen to show an example of how a user would use the toolbox to determine the number of different teleoperation UI configurations required to be designed to teleoperate each individual robot within the robot team.

**Figure 9:**
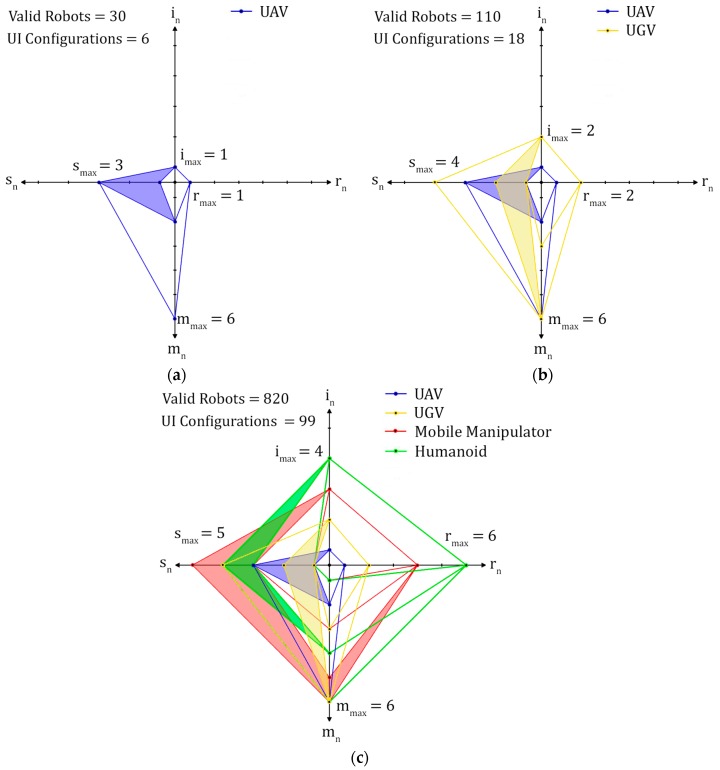
Radar plots for toolbox results for (**a**) Case 1, (**b**) Case 2, and (**c**) Case 3. Shaded areas represent characteristic selections which are numerically continuous.

Case 1 consists of UAVs that may contain 2D, 360°, or RGB-D cameras, direct flight and/or waypoint Robot Motion Control methods, and have up to five onboard sensors. This results in thirty valid robot characteristic combinations with six different UI configurations required to teleoperate each individual robot in the team. As can be observed, for the specified relationship rules, the number of different UI configurations is far less than the number of robot combinations. This is because rather than providing an individual UI for each different valid robot that is represented by each robot characteristic combination as determined by characteristic selections, characteristics are shared as defined by the relationship rules and can therefore be teleoperated with a reduced set of six UI configurations. Case 2 uses the same information as Case 1, with the addition of UGVs that may contain a 2D camera, Light Detection and Ranging (LIDAR), speaker and microphones Sensor Types with a maximum of ten sensors onboard with direct driving and/or waypoint Robot Motion Control methods. This results in 110 valid robot combinations with eighteen different UI configurations. The final case adds Mobile Manipulator and Humanoid Robot Kinematic Types with corresponding characteristics detailed in Table 2, resulting in 820 valid robot combinations and nighty nine different UI configurations. The results for each case are illustrated in Figure 9, with shaded areas representing regions of numerically continuous robot characteristics selections. These results show a significant difference between the number of robot characteristic combinations and UI configurations by sharing common characteristics as discussed throughout this paper.

As demonstrated by these three Cases, the number of different teleoperation UI configurations required to teleoperate the robot team can be determined based on selected robot characteristics required to control possible robot team configurations. These UI configurations represent the number of robot characteristic combinations within the team as reduced using relationship rules. These relationship rules provide users an efficient way to define a systematic approach to teleoperation UI design, reducing the number of teleoperation UI designs as opposed to providing a UI configuration for each individual robot within a team that is typically the case. This approach also provides teleoperators with consistent and familiar UI configurations for different robots from the same Robot Kinematic Type by sharing similar characteristics. This approach improves the teleoperation systems overall interoperability and increases the amount of transferable skills, improving robot switching and situation awareness. Using the toolbox relationship rules also lends itself to later including new robot characteristics that could be introduced by the ROS or robotics community, helping to future-proof against the introduction of new Robot Motion Controls, Sensor Types, or even Robot Kinematic Types characteristics.

## 5. Conclusions and Future Work

This paper proposes an approach relating robot characteristics and teleoperation UI design to help develop a systematic approach to reduce the number of teleoperation UI configuration required to teleoperate each individual robot within a heterogeneous robot teams. A Matlab toolbox was developed allowing users to define relationship rules (informed by ROS metadata and teleoperator experience) between robot characteristics and UI components. The toolbox then identifies a number of different teleoperation UI configurations required for the given robot team based on characteristic selections. The relationship rules reduce the number of teleoperation UI configurations required for a particular team by identifying the same or similar robot characteristics for different robots within the same Robot Kinematic Types. Three test cases are used to show an example of how the toolbox allows users to select robot characteristic for each Robot Kinematic Type; results show the total number of valid robot characteristic combinations and the number of different teleoperation UI configurations required to teleoperate each individual robot within the team.

Proposed future work looks to explore the auto identification of Robot Kinematic Types using ROS URDF metadata and soft computing techniques. Investigation into the representation of motion controls supported by ROS robots is also required. The objective is to propose a standard approach to obtaining robot motion control strategies available on supported ROS robots by using a similar approach to the URDF and SRDF description formats. Other future work includes using common 3D geometry, UI components, and common presentation techniques that could be procedurally generated to automatically build the UI configuration designs required as determined by the Matlab toolbox. This could be used in real-time to automatically assign a different UI configuration to the teleoperator based on their current teleoperation requirements which are supported by the selected robot’s capabilities.

## Figures and Tables

**Table 1:** Definitions defining the robot characteristics used in the Matlab toolbox.

**Robot Kinematic Type** $\left(r_{i}\right)$	
UAV	Flying robot that includes quadcopters, hexacopters, and octocopters [32,33,34].
UGV	Mobile robot that doesn’t contain any manipulators and uses either wheels or special tracks in order to navigate their terrain [35,36].
Manipulator	Replicates an arm represented by a chain of joints between its base and end effector [37].
Mobile Manipulator	A mobile manipulator is any robot that has at least one manipulator and has the ability to move around their environment using a mobile base [38,39].
Torso	A torso robot typically replicates the upper half of a human body; it includes more than one manipulator, and doesn’t have the ability to navigate its environment through the likes of a mobile base [40].
Humanoid	A humanoid robot is one that contains at least two arms, two legs, and a head closely replicating a human being; it may also consist of a waist joint [41].
**Sensor Types** $\left(s_{i}\right)$	
2D and 3D Cameras	Provides limited FoV, 3D cameras provide the added benefit of stereoscopic vision [28].
360° Camera	Provides complete 360° FoV generally overlaid on spherical geometry best viewed using a Head Mount Display (HMD) [29].
Speaker and Microphone	Auditory sensors providing teleoperators the ability to listen and or communicate using sound [30].
Force Sensor	Provide teleoperators force feedback information using a haptic device for physical interactions [31].
2D and 3D Scanning	Provide visual representation of the remote environment using point clouds that can be processed into solid objects and best viewed using a HMD similar to 360° cameras [15].
**Robot Motion Control** $\left(m_{i}\right)$	
Joint	Pure teleoperation used for individual joint control [22].
Flight	Used to fly UAVs as a pure teleoperation with yaw, pitch and roll controls.
Driving	Used to control UGV, mobile bases, etc. typically has backward, forward and turning controls.
Walking	Pure teleoperation method for a teleoperator to control the direction and pace of a given humanoid [42].
End Effector	Used to position the end effectors of manipulators; could be used in combination of object identification to pick and place particular objects [12].
Waypoint	Waypoint provides the teleoperator the ability to select a particular location for example a GPS coordinate on a map; the robot then has the ability to navigate to the point using its own path finding techniques [43].

**Table 2:** Example test cases showing robot characteristic selections for each Robot Kinematic Type.

Case	Robot Kinematic Type	Robot Motion Control	Sensor Types	No. of Sensors
1	UAV	Flight Waypoint	2D Camera 360° Camera RGB-D Camera	5
2	UAV	Flight Waypoint	2D Camera 360° Camera RGB-D Camera	5
	UGV	Driving Waypoint	2D Camera LIDAR Speaker Microphone	10
3	UAV	Flight Waypoint	2D Camera 360° Camera RGB-D Camera	5
	UGV	Driving Waypoint	2D Camera LIDAR Speaker Microphone	10
	Mobile Manipulator	Joint Driving End Effector Waypoint	2D Camera RGD-D Camera LIDAR Speaker Microphone Force Sensor	13
	Humanoid	Joint Walking Waypoint	2D Camera 3D Camera LIDAR RGB-D Camera Speaker Microphone	25
